# Prospective examination of synthetic 5-methoxy-N,N-dimethyltryptamine inhalation: effects on salivary IL-6, cortisol levels, affect, and non-judgment

**DOI:** 10.1007/s00213-019-05414-w

**Published:** 2019-12-10

**Authors:** Malin V Uthaug, Rafael Lancelotta, Attila Szabo, Alan K Davis, Jordi Riba, Johannes G Ramaekers

**Affiliations:** 1 Department of Neuropsychology and Psychopharmacology, Faculty of Psychology and Neuroscience, Maastricht University, Universiteitssingel 40, Maastricht, 6229 ER The Netherlands; 2 Innate Path, Lakewood, CO USA; 3 NORMENT, Institute of Clinical Medicine, University of Oslo, Norway; Division of Mental Health and Addiction, Oslo University Hospital, Oslo, Norway; 4 College of Social Work, The Ohio State University, Columbus, OH 43210 USA; 5 Center for Psychedelic and Consciousness Research, Department of Psychiatry and Behavioral Sciences, Johns Hopkins University, Baltimore, MD 21224 USA

**Keywords:** 5-methoxy-N,N-dimethyltryptamine, Field study, Affect, Immunology, Mindfulness

## Abstract

**Rationale:**

5-methoxy-N,N-dimethyltryptamine is a psychotropic substance found in various plant and animal species and is synthetically produced. 5-methoxy-N,N-dimethyltryptamine is used in naturalistic settings for spiritual exploration, recreation, or to address negative affect and mood problems. However, scientific knowledge on the effects of *5-*methoxy-N,N-dimethyltryptamine in humans is scarce.

**Objectives:**

The first objective was to assess the effects of inhalation of vaporized synthetic 5-methoxy-N,N-dimethyltryptamine on neuroendocrine markers. The second objective was to assess effects of the substance on affect and mindfulness. In addition, we assessed whether ratings of subjective measures were associated with changes in stress biomarkers (i.e., cortisol) and immune response (i.e., IL-6, CRP, IL-1β), as well as the acute psychedelic experience.

**Methods:**

Assessments (baseline, immediately post-session, and 7-day follow-up) were made in 11 participants. Salivary samples were collected at baseline and post-session and analyzed by high-sensitivity enzyme-linked immunosorbent assay (ELISA).

**Results:**

5-methoxy-N,N-dimethyltryptamine significantly increased cortisol levels and decreased IL-6 concentrations in saliva immediately post-session. These changes were not correlated to ratings of mental health or the psychedelic experience. Relative to baseline, ratings of non-judgment significantly increased, and ratings of depression decreased immediately post-session and at follow-up. Ratings of anxiety and stress decreased from baseline to 7-day follow-up. Participant ratings of the psychedelic experience correlated negatively with ratings of affect and positively with ratings of non-judgment.

**Conclusion:**

Inhalation of vaporized synthetic 5-methoxy-N,N-dimethyltryptamine produced significant changes in inflammatory markers, improved affect, and non-judgment in volunteers. Future research should examine the effect of 5-methoxy-N,N-dimethyltryptamineamine with healthy volunteers in a controlled laboratory setting.

## Introduction

Tryptamine psychedelics such as psilocybin are known to alter perception, mood, and a host of cognitive processes (Nichols 2004) and have been indicated for the treatment of mood disorders (Carhart-Harris et al. 2018; Griffiths et al. 2006; Viol et al. 2019). Additionally, classical serotonergic hallucinogens such as LSD or psilocybin are considered physiologically safe and do not produce dependence or addiction (Nichols 2004). A similar potential might exist for the short-acting tryptamine 5-methoxy-N,N-dimethyltryptamine (5-MeO-DMT).

5-MeO-DMT was first synthesized in 1936 (Hoshino and Shimodaira 1936), but can also be found naturally in plants such as the *Dictyoloma incanescens* (Pachter et al. 1959), or in the milky-white secretion, also known as bufotoxin, which is produced in the skin and the parotid glands of the *Incilius alvarius* toad, also known as *Bufo alvarius* (Weil and Davis 1994). A recent lab analysis revealed that about 25–30% of the bufotoxin, used in a naturalistic setting, consisted of the primary component 5-MeO-DMT, as well as (very) low amounts of additional tryptamines such as bufotenine (0.08–0.18%), DMT, and N-methylserotonin (0.01–0.03%) (Uthaug et al. 2019).

5-MeO-DMT acts as a serotonin 5-HT_1A_/5-HT_2A_ receptor agonist (Shen et al. 2010) and is reported to be psychoactive in humans in doses as low as 3–5 mg of pure substance when vaporized (Weil and Davis 1994). 5-MeO-DMT induces a diversity of subjective effects, including auditory, visual, and time perception distortions, emotional experiences, and memory impairment that vary depending on the dose and route of administration (Ott 2001; Shulgin and Shulgin 1997). In fact, low doses of 5-MeO-DMT (bufotoxin source 50 mg, estimated 5-MeO-DMT content 5–7 mg) appear to occasion mystical experiences of similar intensity to high-dose psilocybin (Barsuglia et al. 2018), but with a much shorter duration of action.

Nevertheless, from a recent survey among users in the general population, it appears that 5-MeO-DMT has an acceptable physiological safety profile in naturalistic settings (Davis et al. 2018; Davis et al. 2019), as well as a low addiction liability (Davis et al. 2018). Adverse responses include mental challenges such as feelings of grief, anxiety, panic, or paranoia (J. Barsuglia et al. 2017; Davis et al. 2018). Resurfacing of these experiences can occur even weeks after the experience and may lead to psychological difficulties, particularly in the absence of counseling (Johnson et al. 2019).

At the present time, 5-MeO-DMT has become popular in naturalistic settings as a means for spiritual exploration, recreation, or as a way to increase affect or relieve problems associated with mental health (Davis et al. 2018). Unpublished reports of 5-MeO-DMT use describe inhalation, smoking, or vaporizing as a common route of consumption (Erowid, n.d.). Interestingly, people who reported having had a psychiatric condition described that following the use of 5-MeO-DMT, they experienced improvement in depression (77%), anxiety (69%), post-traumatic stress disorder (79%), and substance use disorders (60%) (Davis et al. 2018). A recent field study (Uthaug et al. 2019) replicated such reports and showed that a single inhalation of vapor from dried toad secretion containing 5-MeO-DMT produces sub-acute and long-term improvements in affect and cognition in 42 undiagnosed participants in naturalistic ceremonies.

The aforementioned research underpins a potential role for 5-MeO-DMT as a therapeutic tool which may be similar to ayahuasca, an Amazonian plant preparation that contains the closely related psychedelic compound DMT (Palhano-Fontes et al. 2018; Uthaug et al. 2018). The potential of ayahuasca to rapidly and persistently reduce symptoms of depression has been shown in a range of open-label studies (de Lima Osório et al. 2011; Sanches et al. 2016) and was confirmed in a randomized placebo-controlled clinical trial in patients suffering from treatment-resistant depression (Palhano-Fontes et al. 2018). DMT and 5-MeO-DMT have also been shown to exert potent anti-inflammatory effects through the modulation of innate and adaptive immune processes (Szabo 2015; Szabo et al. 2014). Moreover, in vitro studies have recently provided evidence of anti-inflammatory, neuroregenerative, and anti-addictive effects induced by 5-MeO-DMT (Dakic et al. 2017). In addition, 5-MeO-DMT has been demonstrated to affect neurogenesis, which may contribute to the known antidepressant properties of DMT-derived compounds (Lima et al. 2018). The potential in vivo anti-inflammatory effects of psychedelic tryptamines may open up novel vistas in the treatment of a wide range of diseases including, but not limited to, various autoimmune and neurodegenerative disorders (Szabo 2015). Furthermore, according to the immune hypothesis of psychiatric diseases, therapeutic control of peripheral and brain inflammation could offer novel, important tools in the treatment of severe mental and mood disorders, such as major depression (Khandaker et al. 2015; Miller and Raison 2016). Thus, therapeutic inflammatory modulation by serotonergic tryptamines (e.g., 5-MeO-DMT) may represent new and effective treatment modalities in a broad spectrum of clinical pathologies.

The present study was conducted to prospectively assess the effects of 5-MeO-DMT on stress and inflammatory immune functions in users that participated in naturalistic 5-MeO-DMT sessions. A secondary aim was to assess the effects of 5-MeO-DMT on depression, anxiety, stress, mindfulness, and satisfaction with life. A third aim was to determine whether changes in mental health variables were related to the intensity of the psychedelic experience and changes in the stress and immune system response. We expected that symptoms of depression, anxiety, and stress would decrease, and ratings of satisfaction with life would increase, from before to after inhalation of vaporized synthetic 5-MeO-DMT. Whether mental health changes produced by tryptamines are also associated with changes in stress and immune response is presently unknown. However, we hypothesized that salivary stress and immune biomarkers would allow further exploration of the association between anti-inflammatory (Szabo 2015; Szabo et al. 2014), and mental health effects related to the inhalation of 5-MeO-DMT (Uthaug et al. 2019).

## Methods

For this observational study, we visited a location in Prague, The Czech Republic, where people inhaled vapor from synthetic 5-MeO-DMT administered by a facilitator in individualized, one-on-one sessions. Participants were invited to enter the current study when they first contacted the facilitator to sign up for the session and gave their written informed consent on location prior to drug administration. A total of 11 participants were approached and consented to participate in the research. These individuals completed baseline and a post-session assessment on location while 10 participants also completed an online follow-up assessment 7 days after intake through Qualtrics, a Web-based survey tool to conduct survey research. Exclusion criteria included non-fluency in English, taking (any) medication and age < 18 years. None of the participants were excluded based on these criteria. Participation was voluntary, and no incentives to participate were provided. The study was approved by the Ethical Review Committee Psychology and Neuroscience (ERCPN), in Maastricht, the Netherlands.

Most participants were from Europe (*N* = 8) and the rest of the participants were from North America (*N* = 3). There were eight males and three females. Mean age of the entire group was 33 years of age (SD = 8.59). Participants had obtained a university bachelor’s degree (*N* = 6), a master’s degree (*N* = 4), or completed a trade school (*N* = 1). All participants reported having previous experience with psychedelic substances. In total, nine participants reported having had no previous experience with 5-MeO-DMT, while two participants reported having had 5-MeO-DMT on three and five previous occasions, respectively. The participants’ motivations to use 5-MeO-DMT included “to understand myself” (*N* = 2), “out of curiosity” (*N* = 2), “solve problems” (*N* = 1), “to understand myself and solve problems” (*N* = 2), “to understand myself and out of curiosity” (*N* = 1), “to understand myself, solve problems and out of curiosity” (*N* = 2), and finally “to understand myself” and “other”, namely “explore possibilities” (*N* = 1). Several participants reported to have not used any substances the past 7 days (*N* = 4), others reported having used alcohol (*N* = 3), cannabis (*N* = 2), alcohol and cannabis (*N* = 1), alcohol and 3,4-methylenedioxymethamphetamine (MDMA) (*N* = 1). No participant reported having received a diagnosis of mental health disorder from a clinician. However, four participants reported past problems related to depression (*N* = 1), depression and anxiety (*N* = 1), depression, anxiety, and post-traumatic stress disorder (PTSD) (*N* = 1), and other (*N* = 1).

### Setting of the sessions

The sessions for each participant were held at a yoga studio in Prague in the Czech Republic. The facilitator was male, trained in Holotropic Breathwork, a practice that uses breathing and other elements to putatively allow access to non-ordinary states of consciousness (Grof and Grof 2010) and had been facilitating sessions using 5-MeO-DMT for about 2 years. For further elaborate details on the setting of the sessions, see supplementary content in Appendix 1.

### Study procedure

In this naturalistic observational study, assessments were taken at baseline, immediately post-session, and at 7 days in participants who inhaled vapor from synthetic 5-MeO-DMT. On location, prior and after their session, participants completed a 15-min test-battery consisting of a demographic section, questionnaires assessing mental health-related factors (depression, anxiety, stress, satisfaction with life and mindfulness-related capacities). In addition, two salivary samples were collected, one 30 min before the participant session (baseline), and post-session (within 1–1.5 h after the session ended). Saliva samples were collected in Saliva Collection Aid (SCA) tubes (Salimetrics, Carlsbad, CA, USA), immediately stored, and transported on dry ice. Finally, 10 out of 11 participants completed the 7-day follow-up assessments, which included mental health measures as distributed at baseline and post-session, as well as questions related to the overall experience.

### Subjective measures of mental health

The test battery consisted of five questionnaires in their original language English; the Depression, Anxiety and Stress Scale 21 (DASS-21), the Satisfaction With Life Scale (SWL), the Five Facets Mindfulness Questionnaire (FFMQ-39), the Ego Dissolution Inventory (EDI), and the 5-Dimensional Altered States of Consciousness Rating Scale (5D-ASC). EDI and 5D-ASC were distributed after the session to assess the experience in retrospect.

#### EDI

EDI is an 8-item self-report scale that assesses the participant’s experience of ego dissolution (Nour et al. 2016). The participants answered the scale with making a mark on a line from either “No, not more than usually” (0%) to “Yes I experience this completely/entirely” (100%). The total EDI is scored by calculating the mean percentage of all the eight items. The higher the total score, the stronger the experience of ego dissolution. The internal consistency of the total scale in the current sample was excellent (Cronbach’s alpha = .909).

#### 5D-ASC

The 5D-ASC is a 94-item self-report scale that assesses the participants’ alterations from normal waking consciousness (Adolf Dittrich 1998; A Dittrich et al. 2010; Studerus et al. 2010). The participant is asked to make a vertical mark on the line below each statement to rate to what extent the statements applied to their experience in retrospect (i.e., from 0% “No, not more than usually” to 100% “Yes, more than usually”). The 5D-ASC measures 11 subscales: experience of unity spiritual experience, blissful state, insightfulness, disembodiment, impaired control and cognition, anxiety, complex imagery, elementary imagery, audio-visual synesthesia and changed meaning of perception and five key dimensions; oceanic boundlessness, anxious ego dissociation, visual restructuralization, auditory alterations, and finally reduction of vigilance. Moreover, the 5D-ASC measures five key dimensions which include oceanic boundlessness that identifies mystical-type experiences and has been compared with the “heaven” aspect of Huxley’s mescaline account (Adolf Dittrich 1998) anxious ego dissociation, visual restructuralization, auditory alterations, and reduction of vigilance. The internal consistency of the total scale in the current sample was excellent (Cronbach’s alpha = .953).

#### DASS-21

DASS-21 is the shorter version of the original self-report questionnaire Depression, Anxiety, Stress Scale 42 (Henry and Crawford 2005). The purpose of the DASS-21 scale is to measure constructs of depression, anxiety, and stress ranging from 0 (normal) to 42 (extremely severe). The participants responded by rating the concordance with each statement from 0 “Did not apply to me at all” to 3 “Applied to me very much, or most of the time”. The sub-scale scores for depression (*α* = .88), anxiety (*α* = .82), and stress (*α* = .90) are calculated by summing the scores for the relevant items to the characteristic being measured (Henry and Crawford 2005). As the original DASS has 42 questions, the sum of the DASS-21 is multiplied by 2 to ascertain the comparable scores. The internal consistency of the total scale in the current sample was excellent (Cronbach’s alpha = .918).

#### SWL

SWL is a 5-item self-report scale, assessing someone’s subjective satisfaction with life (Diener et al. 1985; Pavot and Diener 2009). The SWL has possible score range of 5 to 35, with 5–9 indicating an extreme dissatisfaction with life, while scores between 31 and 35 indicating that the respondent is extremely satisfied. The items are answered on a Likert-scale ranging from 1 “Strongly disagree” to 7 “Strongly agree”. The total score is obtained by adding points on each item. The internal consistency of the total scale in the current sample was good (Cronbach’s alpha = .814).

#### FFMQ

FFMQ is a 39-item self-report scale (Baer et al. 2006) and measures the following five facets of mindfulness-related capacities: (1) Observe: noticing experience that are both internal and external such as thoughts and emotions; (2) Describe: describing internal experiences; (3) Acting with awareness: focus on the present activity; (4) Non-judgment: not evaluating or judging the present experience; (5) Non-reaction: allowing thoughts and feelings to come without acting or reacting upon them. The purpose of this scale is to obtain an understanding of an individual’s mindfulness-related capacities. The participants answered the FFMQ by rating the concordance with each statement on a 5-point Likert-scale that ranges from 1 “never true” to 5 “very often or always true”. Facet scores were computed by summing the scores on the individual items. Facet scores range from 8 to 40 (except for the non-reactivity facet, which ranges from 7 to 35), with higher scores indicating more mindfulness. The internal consistency of the total scale in the current sample was excellent (Cronbach’s alpha = .936).

#### Analysis of saliva samples

All saliva samples (2–3 mL/sample) were assayed for cortisol, IL-6, CRP, and IL-1β using high-sensitivity ELISA kits following the manufacturer’s recommended protocol (Salimetrics). The test volume was 25 μL, with a range of standards from 0.012 to 3.000 μg/dL (cortisol), 0 to 100 pg/mL (IL-6), 93.75 to 3000 pg/mL (CRP), 3.13 to 200 pg/mL (IL-1β), and the assays had a lower limit of sensitivity of 0.007 μg/dL (cortisol), 0.07 pg/mL (IL-6), 10 pg/mL (CRP), and < 0.037 pg/mL (IL-1β). Samples were thawed and immediately centrifuged at 3000 rpm for 15 min to remove mucins and diluted 1:2 (cortisol, IL-6, IL-1β) or 1:10 (CRP) prior to assay. All samples were measured in duplicate, and the average of the duplicates was used in the statistical analyses. The intra- and inter-assay coefficients of variation were less than 10% and 15%, respectively. Absorbance measurements were carried out using a Synergy HT microplate reader (Bio-Tek Instruments, Winooski, VT, USA) at 450 nm.

#### Statistical analysis

A repeated measures ANOVA using a linear mixed model analysis with session, the assessment point (three levels; baseline, post-session, and follow-up) as the within-subject factor was conducted. Fixed main effects included session, using the maximum likelihood method. Covariance structure was chosen according to best fit and included compound symmetry heterogeneous (CSH), unstructured correlations (UNR), as well as heterogeneous first-order autoregression (AR1) structures. Significant main effects of session were followed by separate least significant difference (LSD) contrasts between baseline and follow-up session. The alpha level of significance was set at 0.05. Pearson’s correlations were carried out to investigate the association between the level of ego dissolution, and the experience of altered states of consciousness with subjective measures of affect and cognition. In addition to investigating the association between any of the psychometric data, and the saliva ELISA data, Cohen’s *d* was calculated to estimate effect sizes of significant mental health changes between baseline and post sessions, and Hedges’ g was calculated to estimate effect sizes of significant mental health changes between baseline and follow-up sessions. Eta square scores **(**η_p_^2^) were calculated to estimate effect sizes of significant mental health changes between sessions. The data was analyzed with the Statistical Package for the Social Sciences 24.0 (IBM SPSS, 2016). For the statistical analysis of saliva ELISA data, two groups (“baseline” [collected 30 min before the participants session] and another one “immediately post-session” [collected within 1–1.5 h after their session ended], where the samples of each participants were paired up) were compared with Student’s *t* test. Data analyses were performed using GraphPad Prism version 8.00 for Windows (GraphPad 2019) (GraphPad Software Inc., La Jolla, CA, USA, www.graphpad.com). Differences were considered to be statistically significant at *p* < 0.05.

## Results

### Dose

During their individual session with 5-MeO-DMT, three participants had one dose of 5-MeO-DMT, one participant had two doses, five participants had three doses, and two participants had four doses. The doses varied by participant and ranged from 3 to 24 mg. The summed total doses varied between 17 and 61 mg. Doses were administered between 8 and 25 min apart, and additional doses were given only when discernible effects of the previous dose had diminished. For an overview of dose(s), and inhalation time, see Table 1.

**Table 1:** An overview of mean ratings of ego dissolution (0–100%), the weighed individual dose(s) of synthetic 5-MeO-DMT per participant in milligrams, as well as the average duration of inhalation of vaporized synthetic 5-MeO-DMT and breath held (in seconds)

Participant	Dose 1 (in mg)	Dose 2 (in mg)	Dose 3 (in mg)	Dose 4 (in mg)	Sum of dose (in mg)	Average duration of inhalation (in seconds)	Average duration of breath held (in seconds)	EDI score (in %)
1	6	17	21		44	44.3	25.5	11.88
2	6	19			25	23.5	23.5	68.13
3	7	15	23		45	25.7	10.3	67.5
4	4.60	6	9	12	31.6	31.3	24.5	72.75
5	18				18	15	10	53.13
6	21				21	30	20	76.25
7	17				17	30	34	100
8	3	6	9	14	32	23	15.5	0
9	6	19	23		48	27.7	15.7	50
10	5	14	21		40	27.7	15.3	35.63
11	18	19	24		61	32	6.3	35

### Adverse reactions

Notably, 45.5% (*N* = 5) of the sample reported adverse effects post-session. One participant reported feeling “scared and confused”, one participant reported “feeling anger, joy love and fear”, one participant vomited shortly after intake, one participant expressed “feeling a little shock on the first try, but nothing bad”, and finally one participant reported feeling that their throat was scratching from smoking. On the 7-day follow-up, 27.3% (*N* = 3) of the sample reported adverse effects in the days following the session. One participant reported some affect and somatic tension in muscles, one participant reported difficulties sleeping (insomnia), and finally, one participant reported experiencing somatic tension in muscles, in the 7 days following their session.

### The psychedelic experience

#### EDI

**Fig. 1:**
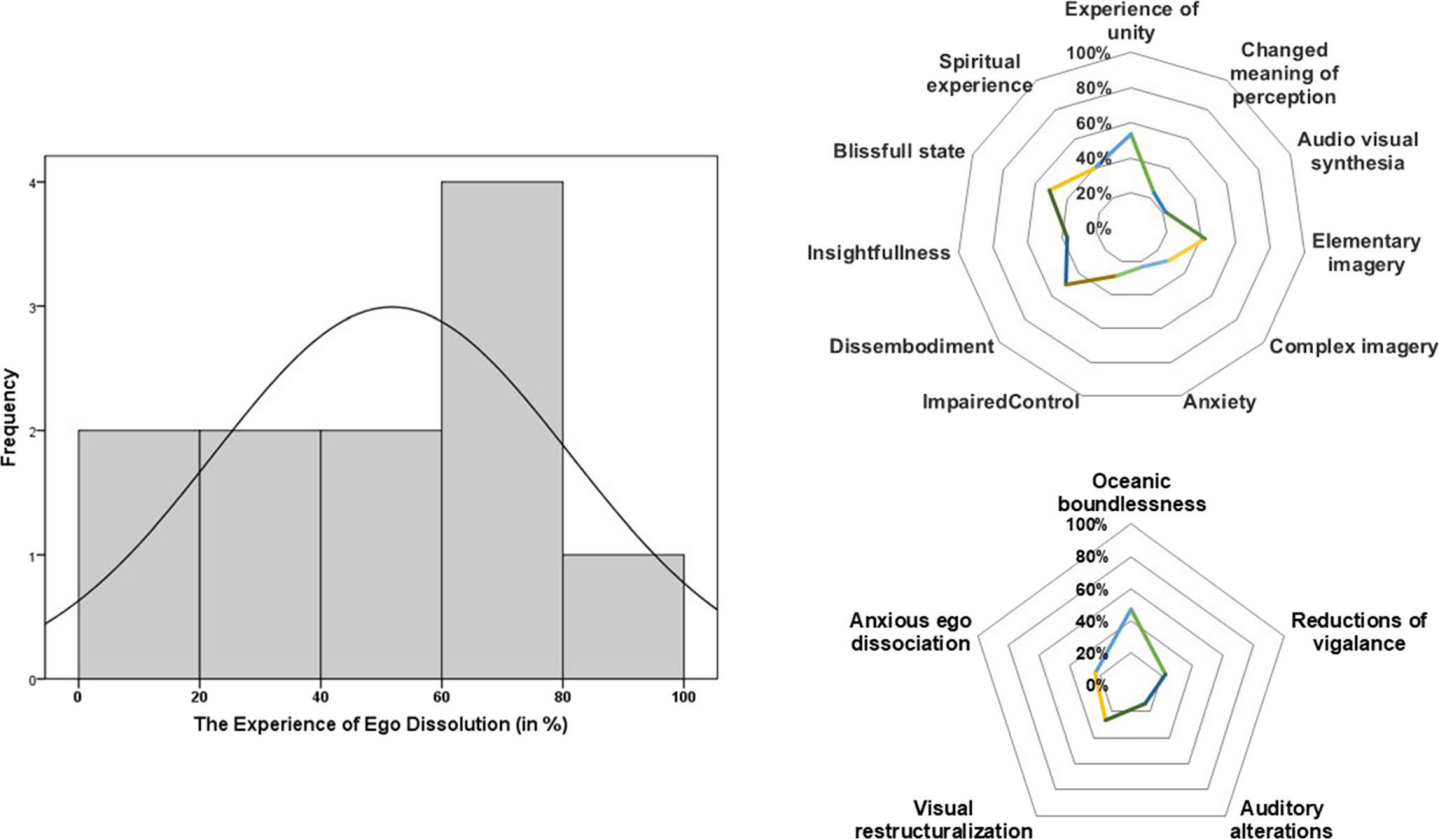
Frequency distribution of the experience of ego dissolution as assessed by the EDI post-session after inhalation of vaporized synthetic 5-MeO-DMT (left panel). Mean ratings (range 0–100%) of the psychedelic experience as assessed with the 5D ASC subscales (upper right panel) and dimensions (lower right panel) after inhalation of vaporized synthetic 5-MeO-DMT

Overall, the mean (SD) ratings of EDI were 51.84% (29.49). A frequency distribution of the EDI ratings is shown in Fig. 1. EDI scores per individual are shown in Table 1.

#### 5D-ASC

The mean (SD) ratings of the experience of altered states of consciousness as rated by the five dimensions of the 5D-ASC were *oceanic boundlessness* = 46.88% (30.11), *anxious ego dissociation* = 23.83% (15.80), *visual restructuralization* = 27.16% (22.76), *auditory alterations* = 14.39% (14.37), and finally *reduction of vigilance* = 22.34% (8.61). Furthermore, the mean (SD) rating of the experience of altered states of consciousness as rated by the 11 sub-scales of the 5D-ASC, ranging from 0 to 100%, is shown in Fig. 1. The top three rated subscales were *experience of unity* 53.82% (36.25), *blissful state* 51.35% (38.94), and *disembodiment* 49.58% (30.84).

### Stress and inflammatory markers

**Fig. 2:**
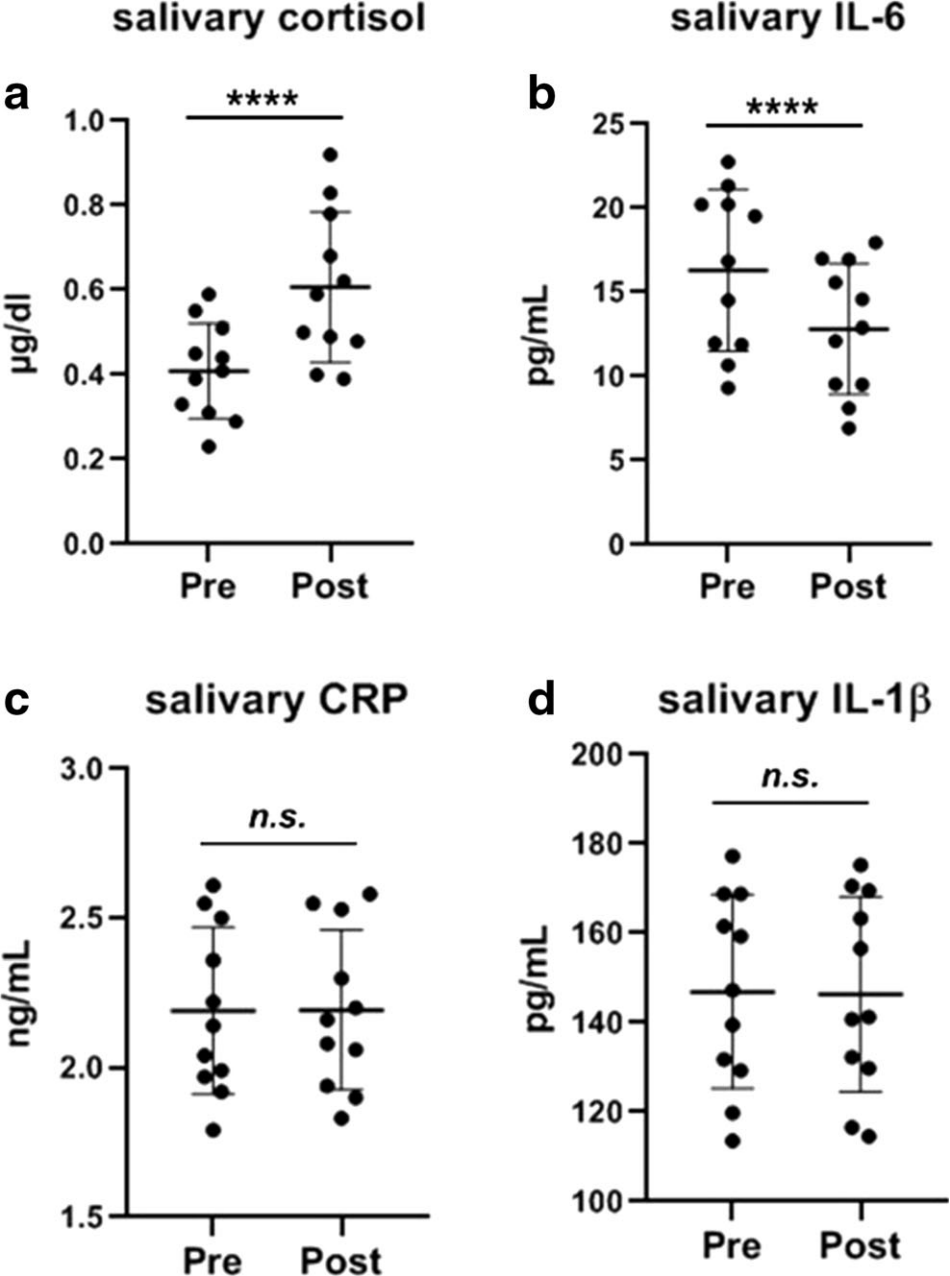
Effect of 5-MeO-DMT on salivary neuroendocrine and immune markers. Saliva samples were collected before (baseline) and 1–1.5 h after (post) the inhalation of vaporized synthetic 5-MeO-DMT and were analyzed by ELISA. Results are shown as mean ± SEM. Asterisks (****) represent *p* values < 0.001; *n.s.* means no statistical significance

Mean difference in (SD) cortisol, CRP, IL-6, and Il-1β salivary concentrations are shown in Fig. 2. Inhalation of vaporized synthetic 5-MeO-DMT resulted in significant increase in the levels of salivary cortisol (mean difference, MD = 0.21 μg/dL; *p* < 0.001) and significant decrease of salivary IL-6 (MD = 3.51 pg/mL; *p* < 0.001). Inhalation of vaporized synthetic 5-MeO-DMT did not produce statistically significant changes in salivary CRP and IL-1β.

The normal/physiological range of the monitored biomarkers in saliva is the following: CRP: <3 ng/mL; cortisol: 0.15–0.65 μg/dL; IL-1β: 50–200 pg/mL; IL-6: 5–25 pg/mL (Desai and Mathews 2014). Baseline levels of all investigated biomarkers were in the physiological range in case of each participant, see Fig. 2.

### Subjective measures of affect and cognition

**Fig. 3:**
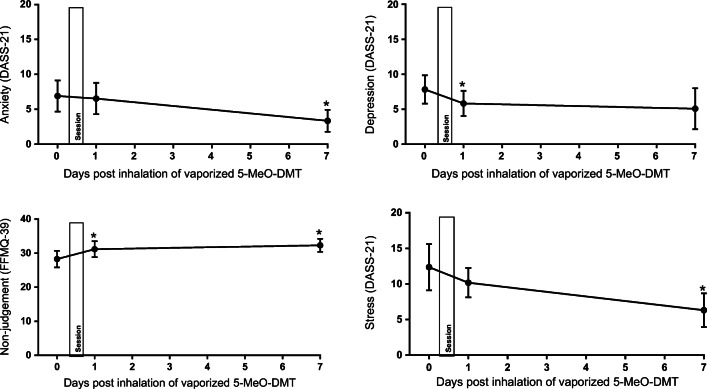
Mean (SE) ratings of anxiety (upper left panel), depression (upper right panel), and stress (bottom right panel) as assessed by DASS-21 (upper panel), and ratings of non-judgment (lower left panel) as measured by FFMQ-39. Before (0), post-session (1), and 7 days after (7) inhalation of vaporized synthetic 5-MeO-DMT (* *p* ≤ .05)

Overall, main effects of session reached significance on anxiety (as assessed by DASS-21) (*F*_2, 19.380_ = 6.023; *p* = .009, η_p_^2^ = 0.38), eta = .551), depression (as assessed by DASS-21) (*F*_2, 9.979_ = 8.104; *p* = .008, η_p_^2^ = 0.18), and non-judgment (as assessed by FFMQ) (*F*_2, 10.182_ = 14.018; *p* = .001, η_p_^2^ = 0.45), while approaching significance in stress (as assessed by DASS-21) (*F*_2, 20.355_ = 3.070; *p* = .068, η_p_^2^ = 0.21), see Fig. 3.

Separate contrasts between baseline and measures immediate post-session revealed a significant reduction in depression ratings (*p* = .049, Cohen’s *d* = 0.31), and a significant increase in non-judgment (*p* = .017, Cohen’s *d* = 0.36). Separate contrasts between baseline and the 7-day follow-up revealed a reduction in stress (*p* = .043, Hedge’s *g* = 0.67), anxiety (*p* = .010, Hedge’s *g* = 0.40), and non-judgment (*p* = .001, Hedges’ *g* = 0.51). Moreover, there was a reduction in depression ratings which approached significance at 7-day follow-up (*p* = .073, Hedges’ *g* = 0.30).

### Correlations

#### Dose

No significant correlations were found between the total dose and measures of the psychedelics experience, salivary biomarker, and subjective measures of affect and cognition. All correlations were very low and ranged from *r* = − .499 to *r* = .240.

### Salivary biomarkers and the psychedelic experience

There were no correlation between mean difference in salivary cortisol and IL-6 (between baseline and immediate post-session) and the ratings of the psychedelic experience (as assessed with EDI and 5D-ASC post-session). Nevertheless, there was a negative correlation between salivary IL-1β and the experience with ego dissolution (*r* = − .679; *p* = .022) and experience of unity (*r* = − .622; *p* = .041). A negative correlation was also found between salivary CRP and oceanic boundlessness (*r* = − .769; *p* = .006), anxious ego dissolution (*r* = − .755; *p* = .007), and auditory alterations (*r* = − .671; *p* = .024), experience of unity (*r* = − .722; *p* = .012), spiritual experience (*r* = − .720; *p* = .012), blissful state (*r* = − .767; *p* = .006), insightfulness (*r* = − 778; *p* = .005), disembodiment (*r* = − .659; *p* = .028), and elementary imagery (*r* = − .670; *p* = .024). Additionally, there was a positive correlation between salivary CRP and anxiety scores (*r* = .664; *p* = .026).

### Salivary biomarkers and subjective measures

There were no correlation between changes in salivary cortisol and IL-6 and ratings of subjective measures between baseline or post-session. However, there was a negative correlation between salivary CRP and awareness (*r* = − .714; *p* = .014) and non-judgment (*r* = − .720; *p* = .012), and a negative correlation between salivary IL-1β and satisfaction with life (*r* = − .874; *p* = .000), and a positive correlation with depression (*r* = .637; *p* = .023).

### The psychedelic experience and subjective measures

**Fig. 4:**
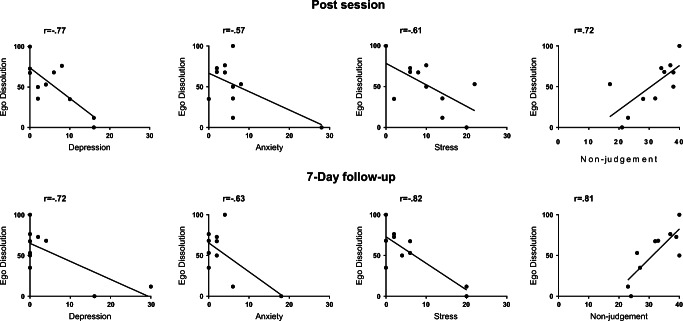
Pearson’s correlations between ego dissolution and affect and non-judgment ratings post-session (upper panel), as well as affect and non-judgment ratings at follow-up (lower panel) after inhalation of vaporized synthetic 5-MeO-DMT

Post ratings of the psychedelic experience (as assessed by EDI and 5D-ASC) were correlated with post and follow-up session ratings of non-judgment (as assessed by FFMQ), depression, anxiety and stress (as assessed by DASS-21), and satisfaction with life (as assessed with SWL). For an overview of correlations between the experience of ego dissolution and subjective measures post-session and 7-day follow-up, see Fig. 4. For an overview of correlations between 5D-ASC ratings and subjective measures, see Table 2 (post-session) and Table 3 (7-day follow-up).

**Table 2.**
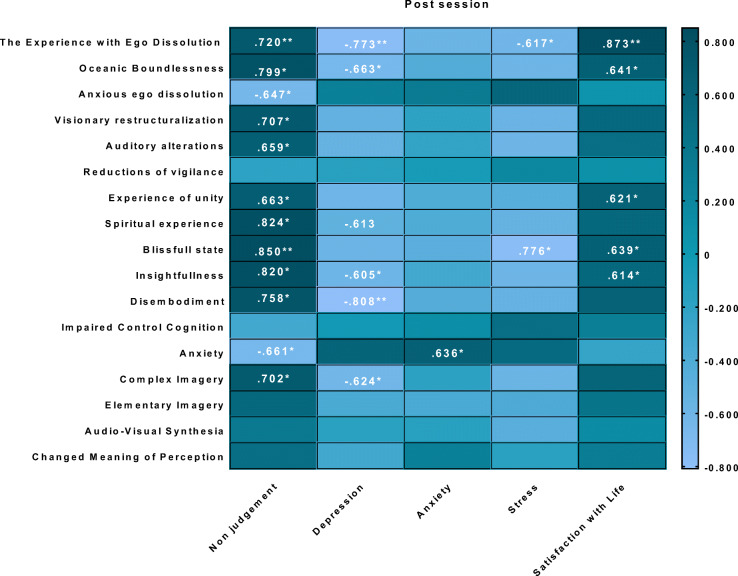
An overview of Pearson’s correlations between the psychedelic experience as assessed by EDI, 5D-ASC (dimensions and subscales) and subjective measures (FFMQ, DASS-21, and SWL), at post-session. *p ≤ .05, **p ≤ .01

**Table 3.**
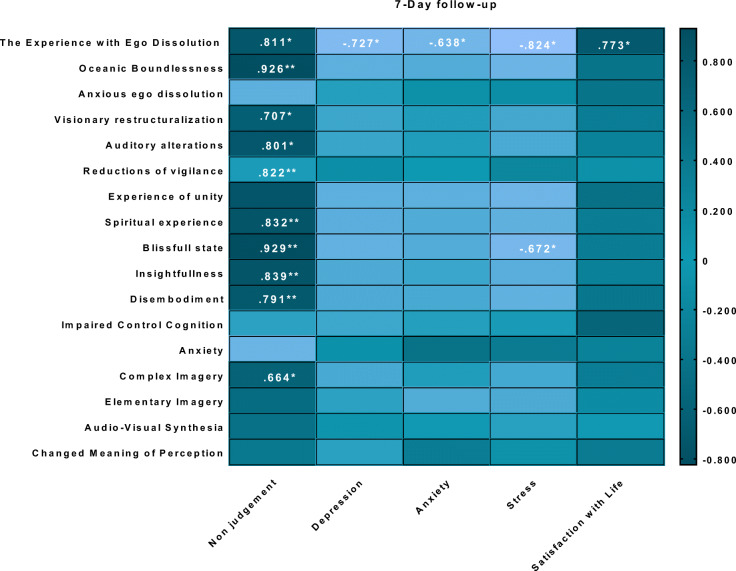
An overview of Pearson’s correlations between the psychedelic experience as assessed by EDI, 5D-ASC (dimensions and subscales) and subjective measures (FFMQ, DASS-21, and SWL), at 7-day follow-up. *p ≤ .05, **p ≤ .01

## Discussion

Participants reported moderate levels of ego dissolution and altered states of consciousness during the psychedelic experience as measured by the EDI and 5D-ASC. These ratings are qualitatively similar but lower in magnitude than reported after inhalation of vaporized toad secretion containing 5-MeO-DMT from the *Incilius alvarius* toad (Uthaug et al. 2019). Inhalation of vaporized synthetic 5-MeO-DMT primarily induced experience of unity, a blissfull state, and elementary imagery whereas inhalation of vaporized toad secretion induced experience of unity, spiritual experience, and a blissfull state (Uthaug et al. 2019). However, in the latter study, the magnitude of ratings was 20–30% higher than in the current study. Likewise, the percentage of individuals that achieved maximum ego dissolution was higher as compared to the present study. These differences in the magnitude of the psychedelic experiences observed may be related to that fact that the sample size in this study was 4 times smaller or to differences in dose. There is little information about 5-MeO-DMT doses that are administered at naturalistic ceremonies, but in the current study, we were actually able to weigh doses before administration. The total dose administered to participants varied widely between 15 and 61 mg and depended largely on personal desires of the participant. However, the magnitude of their psychedelic experience was not significantly correlated the total dose of 5-MeO-DMT that participants received. The current data thus seems to suggest that any dose of 5-MeO-DMT can elicit a psychedelic experience but at an unpredictable magnitude. Other factors causing individual differences may have co-influenced the strength of the experience such as actual drug concentrations in blood, drug metabolism, openness to the psychedelic experience, inhalation technique, or levels of dissociation in the nervous system. In the present study, one participant placed great efforts in resisting the psychedelic experience mentally during four subsequent dose sessions and eventually reported minimal levels of altered states of consciousness.

Five participants (45.5%) reported adverse effects during the session, and three participants (27.3%) reported adverse effects during the days following intake. Adverse events included physical distress and feelings of anger, fear, panic, and sadness. Similar percentages and types of adverse events have been reported in a recent survey among 515 users of 5-MeO-DMT (Davis et al. 2018) where 37% of the responders reported challenging psychological and physical experiences, and 40–60% of respondents reported physical distress or feelings of fear, grief, insanity, isolation, and death. The intensity of these experiences by survey responders, however, was rated as very small. This suggests that adverse events from the psychedelic experience are tolerable but challenging and occur in a sizeable fraction of 5-MeO-DMT users.

Because classic serotonergic psychedelics have been shown to modulate immune responses by downregulating inflammatory processes through 5-HT_2_ and sigma-1 receptors (Nau Jr et al. 2014; Nau Jr et al. 2013; Szabo et al. 2014), we hypothesized that the administration of vaporized synthetic 5-MeO-DMT would alter the levels of inflammatory cytokines and the neuroendocrine-immune regulator cortisol. Interestingly, out of the four salivary biomarkers monitored in this study, only cortisol and IL-6 levels were affected by 5-MeO-DMT administration, see Fig. 2. Cortisol has been widely known to be strongly induced by physical and psychological stress and has an important role in acute stress adaptation, as well as in restoring homeostasis following stressful events (Malarkey and Mills 2007). Modulation of 5-HT_2_ receptors by peak dose(s) of 5-MeO-DMT may represent or mimic an intense neuroendocrine stress-like state; thus, our results showing elevated levels of salivary cortisol are not surprising given the fast-action of inhaled tryptamines and are in good agreement with the literature regarding their fast-acting, stress-mimicking neuromodulatory effects on human physiology (Do Yup Lee and Choi 2015).

We also found that IL-6 was affected by 5-MeO-DMT administration. IL-6 is an important inflammatory cytokine that is released in the early phase of infections and essential in immune defense, but is also involved in pathological autoinflammatory processes (Jones and Jenkins 2018). Pharmacological manipulation of IL-6 secretion or the IL-6 trans-signaling pathway may offer us novel possibilities for the therapy of autoimmune diseases, infections, as well as new generation vaccine design (Garbers et al. 2018). Cortisol and other glucocorticoids are known to rapidly suppress inflammation and have been widely used in the therapy of acute and chronic inflammatory states (Gleeson et al. 2011). Thus, the observed decrease in salivary IL-6 levels may be the direct consequence of increased cortisol secretion independent of any psycho-neuroimmune feedback effects, or 5-HT_2A_ and sigma-1 receptor modulatory actions of 5-MeO-DMT. Furthermore, the in vivo biological half-life of IL-6 is approximately 2 h (Baran et al. 2018), which may explain the observed, relatively low, effect size of IL-6 modulation by 5-MeO-DMT (Fig. 2b). Since salivary and plasma concentrations of the evaluated biomarkers are closely correlated, at least within the context of inflammatory and stress markers, saliva has been suggested as a potential qualitative surrogate for plasma in biomarker studies (La Fratta et al. 2018). Although the sample and effect sizes are low, our results suggest a possible anti-inflammatory effect of 5-MeO-DMT in humans. These findings warrant further investigation into the potential systemic anti-inflammatory effects of 5-MeO-DMT and other DMT analogs.

Although there was no correlation between salivary cortisol and IL-6 and subjective measures and the psychedelic experience post-session or at follow-up, we observed an inverse correlation between CRP levels and both awareness, non-judgment, and several 5D-ASC dimensions post-session, with a positive correlation between anxiety ratings as rated per the 5D-ASC. At follow-up, there was a correlation between CRP levels and both awareness and nonjudgment. Moreover, we also observed an inverse correlation between IL-1β and satisfaction with life and ratings of ego dissolution, and a positive correlation with depression and 5D-ASC ratings, post-session. A negative correlation was found between IL-1β and description and satisfaction with life, and a positive correlation with stress at follow-up. CRP is considered a reliable biomarker of systemic inflammation and inflammatory states. In psychoneuroimmunology and biological psychiatry, inflammation has been tightly linked to neurocognitive processes and behavioral traits (Bauer and Teixeira 2018; Raison and Miller 2011). Systemic inflammation can greatly influence mood, cognitive processing, and psychosocial functioning. For example, it has been shown that inflammatory challenge can increase self-reported feelings of social disconnection and self-awareness (Eisenberger et al. 2010; Moieni et al. 2015). Our CRP-awareness and CRP-non-judgment correlation results are in line with previous findings showing an inverse correlation between CRP levels and psychological parameters (Asakura et al. 2015). Although inhalation of 5-MeO-DMT did not alter CRP levels significantly, CRP is an acute phase protein which production is under the direct control of IL-6, and both have been linked to neuropsychological regulatory processes within the context of inflammation (Del Giudice and Gangestad 2018). We hypothesize that the observed subacute effect of 5-MeO-DMT on salivary IL-6 may also be expanded to significant changes in circulating CRP levels in the long run. Thus, the involvement of the IL-6-CRP-brain axis in the biopsychological effects of 5-MeO-DMT administration in human warrants future research.

Inhalation of vaporized synthetic 5-MeO-DMT produced significant improvements in mindfulness rating of non-judgment and reduced symptoms of depression directly after the session as well as at the 7-day follow-up. Moreover, inhalation of 5-MeO-DMT reduced symptoms of anxiety and stress from baseline which reached significance at the 7-day follow-up. It should be highlighted that the ratings in affect (depression, anxiety, and stress) were subtle which introduces the possibility that the reduction in affect can be interpreted as betterment in well-being. However, the present findings are in line with previous research on use of synthetic 5-MeO-DMT in ceremonial settings (Davis et al. 2019), as well as a naturalistic observational study investigating the effects following use of toad secretion containing 5-MeO-DMT (Uthaug et al. 2019). Additionally, prior research showed that consumers of ayahuasca displayed significant reductions in ratings of depression and stress that persisted for 4 weeks after intake (Uthaug et al. 2018). Likewise, high-dose psilocybin produced large decreases in clinician- and self-rated measures of depressed mood and anxiety, along with increases in quality of life in cancer patients (Griffiths et al. 2016). The authors reported that at the 6-month follow-up, these changes were maintained, with about 80% of participants continuing to show clinically significant decreases in depressed mood and anxiety (Griffiths et al. 2016). Finally, another study that compared aspects of mindfulness before, and 24 h after an ayahuasca session, found that ayahuasca increased mindfulness-related capacities such as non-judgment and non-reaction following intake (Soler et al. 2016). With this in mind, the present result suggests that inhalation of vapor from synthetic 5-MeO-DMT can bring about improvements in affect and aspects of mindfulness that might alleviate symptoms of mood disorders (Baer 2003). These data suggest that clinical research examining the potential therapeutic effect of 5-MeO-DMT is warranted.

Changes in the ratings of subjective measures that were observed in the present study are not necessarily related to a pharmacological effect of 5-MeO-DMT, but could have resulted from uncontrolled variables such as response expectancy of the participants prior to and after the session or the set and setting of drug administration (Carhart-Harris et al. 2018; Hartogsohn 2016). Yet, the finding that the strength of actual psychedelic experience was positively correlated to changes in non-judgment and negatively correlated with symptoms of depression, stress and anxiety strongly suggest that the inhalation of 5-MeO-DMT contributed to changes in these subjective measures. This finding is in line with previous studies on psilocybin; ayahuasca and toad secretion have shown that stronger psychedelic experiences are associated with larger changes in subjective health outcomes (Bogenschutz et al. 2015; Griffiths et al. 2006; Roseman et al. 2018; Ross and Thomas 2010; Uthaug et al. 2019; Uthaug et al. 2018). Still, the present findings are not without limitations. In fact, the small sample size, the possibility that volunteers were biased and thus responded in a social desirable manner as well as the lack of a placebo control group limit the generalizability of the data. The present study should be replicated in a larger sample under experimental, placebo-controlled conditions for a final confirmation.

In conclusion, this prospective study in volunteers showed that inhalation of vaporized synthetic 5-MeO-DMT induced rapid changes in inflammatory markers and produced improvements in affect and non-judgment that lasted for 7 days. The present findings warrant further investigation into anti-inflammatory and mental health effects of synthetic 5-MeO-DMT in clinical trials.
